# Bibliographical Notices

**Published:** 1849-11

**Authors:** 


*Report on the Practical operation of the Law relating to the Importation of adulterated and spurious Drugs, Medicines, &c.* By M. J. Bailey, M. D., Special Examiner of that class of merchandise in the United States Customs at the Port of New York. Read before the New York Academy of Medicine, June 6th, 1849. Published by order of the Academy. From the New York Journal of Medicine. 8vo. pp. 20. New York, 1849.

This report gives us the first official information in regard to the effects of the recent law on the inspection of foreign articles of the materia medica. It had long been known, to those engaged in pharmacy more especially, that the most spurious articles had been introduced extensively into the country; that the galenicals were frequently anything but what they purported to be, and the chemicals not unfrequently most carelessly, and often fraudulently prepared; nay, that positive danger—as in our own experience—had followed the administration of what ought to have been entirely innoxious. Yet few had anticipated such astounding disclosures as are promulged in the present Report; and it must be not a little gratifying to Dr. Edwards, of Ohio—by whom the bill "relating to the Importation of adulterated and spurious Drugs, Medicines, &c.," was brought before, and successfully carried through, Congress—to find that such signal benefits had resulted to the profession of which he is a respected member by his well directed and well crowned endeavours.

We are free to confess, that we had serious apprehensions in regard to the mode in which the law would be carried into effect; and these apprehensions have, in some cases, been verified by the results. There was danger, it appeared to us, that the office of inspector might be bestowed, for political considerations, upon physicians who had not applied themselves especially to the study of pharmacy; or on pharmaceutists whose investigations had not extended farther than might be necessary for keeping a respectably ordered retail establishment. There was danger, too, that the claims of analytical chemistry, as a part of the qualifications of the inspecting officer, would be overlooked; yet it is obvious, that for the detection of spurious or adulterated chemicals it is indispensable; and, accordingly, at an early period, we ourselves took the liberty of urging, in the proper quarter, serious attention to this point.

The Report before us had its origin in the following manner. On the passage of the bill prohibiting the importation of spurious and adulterated drugs, medicines, medicinal preparations, &c., [we should like to see the synonymy of these various terms,] Dr. Bailey was requested "by the Hon. Thomas O. Edwards, the able and philanthropic chairman of the special committee of the House, to which that important subject had been referred, to note the practical working of the law at the port of New York, (where three-fourths or more of the importations of that class of merchandize were presented for entry,) and furnish, in such a manner as might be agreeable to himself, a report of the same, prior to the next meeting of the National Medical Association, in order that the information therein contained might have the greatest possible circulation through the country." p. 3.

The law took effect at the port of New York on the 12th of July, 1848, and the following is a list of the more prominent "drugs and medicines," with the quantities and place whence imported annexed, which Dr. Bailey, during the months named, rejected under its provisions.

| | | | |
|---|---|---|---|
| July, 1848, | 7,581 lbs. | Rhubarb root, | from Canton. |
| August, | 750 lbs. | Opium, | do. Marseilles. |
| do. | 2,940 lbs. | Jalap root, | do. Tampico. |
| do. | 2,249 lbs. | Rhubarb root, | do. London. |
| September, | 646 lbs. | do. do. | do. do. |
| do. | 1,414 lbs. | Gamboge, | do. do. |
| do. | 545 lbs. | Rhubarb, | do. Hamburg. |
| do. | 1,400 lbs. | Senna, | do. Leghorn. |
| do. | 2,900 lbs. | Spurious Yellow Bark, | do. Bordeaux. |
| do. | 875 lbs. | Rhubarb, | do. Canton. |
| do. | 758 lbs. | Opium, | do. London. |
| do. | 1,783 oz. | Iodine. | do. do. |
| do. | 1,075 lbs. | Rhubarb. | do. Marseilles. |
| do. | 4,275 lbs. | Jalap, | do. Vera Cruz. |
| October, | 788 lbs. | Rhubarb, | do. London. |
| do. | 227 lbs. | Myrrh, | do. do. |
| do. | 13,120 lbs. | Spurious Yellow Bark, | do. Maracaibo. |
| do. | 1,875 lbs. | do, do. do. | do. Bordeaux. |
| November, | 412 lbs. | Myrrh, | do. London. |
| do. | 1,280 oz. | Iodine, | do. Glasgow. |
| do. | 860 lbs. | Opium, | do. Smyrna. |
| do. | 185 lbs. | Rhubarb, | do. London. |
| December, | 156 lbs. | Opium, | do. do. |
| do. | 1,065 lbs. | Myrrh, | do. do. |
| do. | 12,800 lbs. | Spurious Yellow Bark, | do. Santa Martha. |
| do. | 392 lbs. | Jalap, | do. Vera Cruz. |
| January, 1849, | 1,300 lbs. | Pectoral Paste, | do. San Juan. |
| do. | 2,071 lbs. | Rhubarb, | do. London. |
| do. | 3,550 lbs. | Jalap, | do. Havanah. |
| do. | 1,930 lbs. | Spurious Bark, | do. Antwerp. |
| February, | 974 lbs. | Rhubarb, | do. London. |
| do. | 1,992 oz. | Iodine, | do. do. |
| March, | 1,104 oz. | Croton Oil, | do. do. |
| do. | 4,894 lbs. | Senna, | do. do. |
| do. | 1,345 lbs. | Spurious Bark, | do. do. |
| do. | 404 lbs. | Opium, | do. do. |
| do. | 1,150 lbs. | Valerian root, | do. Paris. |
| April, | 425 lbs. | Opium, | do. London. |
| do. | 1,273 lbs. | Myrrh, | do. do. |
| do. | 550 lbs. | Jalap, | do. Vera Cruz. |
| do. | 816 lbs. | do. | do. Tampico. |
| do. | 1,450 lbs. | Sarsaparilla, | do. do. |
| do. | 600 lbs. | Spurious Bark, | do. Barranquilla. |

These, with smaller quantities of sundry articles rejected by him from time to time, make the whole amount recorded up to the time of writing his report about *ninety thousand pounds!!*

It would seem, that the mere passage of the law had exerted a salutary effect on foreign importations of chemicals; for, according to Dr. Bailey, no medicinal chemical preparations of any importance have been presented to entry at the port of New York since it took effect. "The agitation of the question for several months prior to the passage of the bill," he observes, "doubtless had the effect of putting those engaged in the nefarious traffic on their guard; and as this country was the principal market for merchandise of that description, it is to be presumed, that the manufacture abroad has been, in a great measure, discontinued. The worthy popular foreign manufacturing chemists, who have never been known to send out from their laboratories an impure article, look, as far as I can learn, upon the law in question with much favor; and well they may, for their sales have, in consequence, greatly increased. This, however, is very readily accounted for from the fact, that by stopping the importation of base adulterations and imitations, the genuine foreign articles are immediately in greater demand, for the reason, that the business of preparing the extensive variety of chemical preparations used in medicine is still in its infancy in this country." p. 9.

Were there no other result flowing from the operation of the new law than that pointed out by Dr. Bailey in the preceding extract, the profession and the public ought to look upon it with great interest, and to use every exertion to have its beneficial operation extended. Not many years ago, we were surprised to find several patients affected with severe vomiting and diarrhœa from an ordinary dose of the harmless *Hydrargyrum cum Cretâ*, imported from abroad from a well known establishment in London; and on examination it was found to be adulterated with the red oxide of mercury!

But although eminent advantage has resulted in the case of foreign importations, Dr. Bailey does not deny that an evil has sprung up as regards domestic manufactures.

He shows, that "as far as sophisticated chemical and medicinal preparations are concerned, we have now but little to fear from the foreign manufacturer and speculator, while we have *increased* cause for vigilance at home, not only as respects the finer preparations, but also that almost equally important class of medicines known to the trade as crude drugs, to wit: opium, barks, roots, medicinal gums, &c. &c., whether in a natural or powdered state:" and he remarks, that of spurious and worthless cinchona barks he has thus far rejected about *thirty-four thousand pounds*, for the reason, that they contained *none*, or but a trace of the natural alkaloids of the *true* barks, and were, therefore, totally devoid of any antiperiodic virtue. "To suppose," says he, "that we have none among us engaged, or who *will* engage in the preparation and sale of spurious and adulterated medicines, is to place a higher estimate upon the conscientious scruples of that portion of our speculating and trading community with whom the almighty dollar is paramount to all other considerations, moral, if not divine, than from my somewhat extensive observations I am willing to concede. Even at this early day, the fraudulent work of their hands is but too visible. Within the last month or two, sulphate of quinine [we wish Dr. Bailey would use the *pharmacopœial* name] "in considerable quantities, bearing the label of Rosengarten and Denis, Philadelphia, has been found in market adulterated to the extent of some twenty or twenty-five per cent. The same may be said of quinine bearing the label of the London Alkaloid Company, likewise that bearing the label of Pelletier, Delondre, and Levailant, Paris. Now these frauds were perpetrated by our own people, or among our own people, and after the article, too, had come into the hands of the purchaser. The manufacturers sent them forth pure, and had nothing to do with the sophistication. Each of the firms named stand [stands] too high, and deservedly so, to warrant even a suspicion of such unpardonable baseness." p. 10.

To prevent these home frauds Dr. Bailey suggests, that the National Medical Association—which has already a committee on the subject of the falsification and adulterations of drugs, of which Professor Huston, of this city, is chairman—"should appoint a committee composed of *two* or more from each State, whose duty it shall be to closely scrutinize powdered drugs, and all other medicinal preparations found on sale throughout the country; and of those inspected, let them purchase small quantities, and subject the same to analysis; and if they prove to be of inferior strength, or to have been fraudulently prepared, let the fraud be promptly exposed through the columns of our numerous medical and other journals; and let the committee report all particulars at the next annual meeting of the Association."

Such a committee could not possibly—we think—reach the evil. What, for example, could two persons, so appointed, accomplish in the Empire State, with her numerous populous cities! The blot, moreover, appears to us to be on the escutcheon of the pharmaceutists, and it behooves them to remove it. If they would institute a proper pharmaceutical police, they could soon, by proper exposure, purge their body of unworthy members, who, from gross venality, and utter recklessness of human life and honesty, are guilty of these nefarious practices. Much, however, has been done by awakening attention to the subject, and we hope to see them abandoned at home, as they have already been by the foreign manufacturers and merchants, owing to the publicity given to them, and the enactment of the wholesome law, under which Dr. Bailey has proved himself so able and energetic an officer.

*A Treatise on the Diseases and Special Hygiene of Females.* By Colombat de l'Isere. Translated from the French, with additions, by Charles D. Meigs, M. D., Professor of Midwifery and the Diseases of Women and Children in Jefferson Medical College, Philadelphia, &c., &c. A new edition revised, with wood cut illustrations. Philadelphia, Lea & Blanchard. 1850.

The early call for a new edition of a work is always convincing proof of its favor with the public, and such is manifestly the case with the work before us. In our notice of the first edition, occasion was taken to speak in terms of high commendation both of the work itself, and of the additions derived from the vast experience of Professor Meigs. To the second, although greatly emended, no new matter has been added, the translator referring the reader to his Letters on Diseases of Females, and his work on Obstetrics, both recent publications, prepared and published since the issue of the first edition of Colombat, in which his opinions are expressed more at large.

Whatever may have seemed exceptionable in style or language in the previous edition, has been thoroughly remodelled in this, and the work is now presented to us in a guise as attractive as any that has ever emanated from the same source.

